# Effects of nest type and sex on blood saccharide profiles in Humboldt penguins (*Spheniscus humboldti*): Implications for habitat conservation

**DOI:** 10.1371/journal.pone.0233101

**Published:** 2020-05-21

**Authors:** David J. Schaeffer, Jeffrey M. Levengood, Michael J. Adkesson

**Affiliations:** 1 Department of Veterinary Clinical Medicine, College of Veterinary Medicine, University of Illinois at Urbana-Champaign, Urbana, Illinois, United States of America; 2 Illinois Natural History Survey, Prairie Research Institute, University of Illinois at Urbana-Champaign, Champaign, Illinois, United States of America; 3 Chicago Zoological Society, Brookfield Zoo, Brookfield, Illinois, United States of America; Charles University, CZECH REPUBLIC

## Abstract

Reproductive success of endangered Humboldt penguin (*Spheniscus humboldti*) colonies in Peru has been associated with nesting habitat type, presumably due to differences in environmental exposure and activity patterns that may affect energy demands and metabolism. Gas chromatography and mass spectrometry were used to determine serum concentrations of 19 saccharides from 30 Humboldt penguins nesting at Punta San Juan, Peru in order to evaluate differences in metabolic state between penguins nesting in a sheltered burrow or crevice (n = 17) and those in exposed surface nests (n = 13). Univariate and multivariate statistical analyses identified serum saccharides (arabinose, maltose, glucose-6-phosphate, and levoglucosenone in particular) that were nest-dimorphic with substantial differences between surface- and sheltered-nesting penguins. Four sugars (arabinose, xylose, fructose-6-phosphate, and sucrose) had ≥ 2-fold difference in concentration between nest types. Seven saccharides were in the top five subsets generated by discriminant analysis; four of these are simple sugars (D-glucopyranose, α ⇄ β; D-glucose; D-maltose; and D-mannose) and three are derivatives (glucose 6-phosphate, levoglucosenone, and N-acetylglucosamine). D-ribose had the highest information values (generated from weight-of-evidence values) followed by glucose 6-phosphate, levoglucosenone, and D-galactose. Sex was not a significant predictor of saccharide concentration. Levoglucosenone, which is a metabolite of the environmental contaminant levoglucosan, was significantly higher in surface-nesting penguins, reflecting a higher rate of exposure in non-sheltered penguins. Differences in the saccharide profiles of surface- and sheltered-nesting Humboldt penguins likely reflect increased metabolic requirements of surface-nesters at Punta San Juan. Conservation of appropriate sheltered-nesting habitat for penguins is essential for sustained reproductive success and colony health.

## Introduction

The Humboldt penguin (*Spheniscus humboldti*) inhabits the southern Pacific coast of South America. Population declines have resulted in the species being classified as “vulnerable” by the International Union for Conservation of Nature [1] and “threatened” by the United States Fish and Wildlife Service [2]. The Humboldt penguin is listed on Appendix I of the Convention on International Trade in Endangered Species of Wild Fauna and Flora [3] and numerous conservation efforts are underway attempting to recover wild populations.

In Peru, the breeding season typically extends from March to December with penguins generally producing two clutches of paired eggs [4]. Central place foragers such as colonial seabirds face considerable energetic constraints traveling between rookery and foraging areas, which can affect reproductive success. As in most penguins, Humboldt penguin pairs share parental duties with roughly equal investment and foraging behaviors are similar between sexes with relation to time and effort [5, 6]. Sex-specific foraging strategies have developed in both mono- and dimorphic species to maximize reproductive success. In monomorphic species that share parental duties, differential sex-specific foraging behaviors or investments can exist, which may result in variances in nutritional requirements and energy metabolism at specific breeding stages. Similar to sex-specific foraging patterns, the type of nesting habitat can lead to differences in environmental exposure and activity patterns that are presumed to affect energy demands and metabolism.

Reproductive colonies of Humboldt penguins in Peru are closely associated with other seabird rookeries, where penguins dig burrows into surface accumulations of dried guano as their preferred nesting habitat. Large-scale commercial harvesting of guano throughout the 19^th^ and 20^th^ centuries resulted in significant loss of nesting habitat, disruption of breeding seasons, and direct death of adult penguins and eggs. Overexploitation of forage fish by commercial fisheries and El Niño-Southern Oscillation (ENSO) events caused a concomitant reduction in major seabird populations responsible for coastal guano production. Cumulatively, these practices resulted in a substantial decrease in the overall population size of the species, as well as disruption of nesting habitats [7]. The loss of traditional burrowing habitat has resulted in many penguins nesting in exposed surface locations, where reproductive success is lower in several *Spheniscus* species due to a less stable microclimate and higher predation risks [8–11]. Burrow nests along cliff tops are the most successful nest type for Humboldt penguins [8].

With the global population of mature Humboldt penguins averaging around 32,000 individuals, the continued protection of breeding colonies and appropriate habitat is paramount. Understanding the impact of nesting habitat on energy demands and reproductive success helps guide sound conservation policy development.

Using 12 serum metabolites (fatty, organic and amino acids), most of which are involved in lipid and carbohydrate metabolism, the sex of 29/30 Humboldt penguins was able to be correctly classified [12]. That research proposed that sex-related differences in serum metabolites during the breeding season were involved primarily in lipid and carbohydrate metabolism that could be important to survival in times of environmental stress [12]. Building upon that research, we aimed to evaluate sex-related differences in concentrations of a suite of sugars and sugar metabolites (collectively, saccharides). Furthermore, the present study aimed to determine if differences in energy utilization between penguins nesting in exposed surface locations and those using sheltered locations (crevices, burrows) reflected the same saccharides. Based on known differences in reproductive success related to nest type and presumed associated differences in energetic demands, we hypothesized saccharide profiles would differ between nest types. However, based on monomorphic foraging patterns, profiles would not differ due to sex.

## Methods

Blood was collected from 30 adult Humboldt penguins in 2009 (May 29 to June 2) during their breeding season as part of a population health assessment project at the Punta San Juan (PSJ), Marine Protected Area, Ica, Peru (15°22'S, 75°12'W) using previously described collection methods authorized under Peruvian permit 131-2009-AG-DGFFS-DGEFFS and by the Saint Louis Zoo Institutional Animal Care and Use Committee (Protocol Number 09–02) [13]. Penguins were nesting in guano or sheltered rock crevices (burrow) or in exposed surface (surface) nests. A 20-gauge needle was used to collect up to 24 mL of blood from the jugular vein for multiple concurrent projects. Blood was placed into serum separator tubes (Vacutainer, BD, Franklin Lakes, New Jersey, USA) and immediately placed in a cooler with ice packs. Serum was separated by centrifugation at 2,300 g for 10 min within 6 hours of collection, placed into cryovials (NUNC, Thermo Fisher Scientific, Rochester, New York, USA), and promptly frozen to -20°C. Within 1–5 days, samples were placed in liquid nitrogen for export and subsequently maintained at -70°C until laboratory analysis. All penguins were examined by a veterinarian and determined to be in normal health based on physical examination, hematology, and plasma biochemistry parameters.

Our analytical methods closely followed Jiye et al. [14] and are detailed in Levengood et al. [4]. Briefly, serum (400 μL) samples were vortexed with 1.5 mL of methanol:water:chloroform (2:1:1) and centrifuged at 11,000 g for 10 minutes. Polar and non-polar phases were collected separately and dried under N_2_. For metabolic profiling, dried polar extracts were derivatized with 80 μL methoxyamine hydrochloride (20 mg mL^-1^) for 60 minutes at 50°C, then with MSTFA (80 μL) at 70°C for 120 minutes, followed by a 2-hour incubation at room temperature. Ten microliters (10 μL) of the internal standard (hentriacontanoic acid, 10 mg/mL) was added to each sample prior to derivatization. Samples were analyzed on a GC/MS system consisting of an Agilent 7890 gas chromatograph, an Agilent 5975 mass selective detector, and an HP 7683B autosampler (Agilent Inc, Palo Alto, California, USA). Gas chromatography was performed on an HP-5MS (60 m × 0.25 mm I.D. and 0.25 μm film thickness) capillary column (Agilent Inc, Palo Alto, California, USA).

The spectra of all chromatogram peaks were compared with electron impact mass spectrum libraries NIST08 (NIST, MD, USA), W8N08 (Palisade Corporation, New York, USA), and a custom-built library. To allow comparison between samples, all data were normalized to the internal standards in each chromatogram and the bird’s weight. The spectra of all chromatogram peaks were evaluated using the HP Chemstation (Agilent Inc, Palo Alto, California, USA) and AMDIS (NIST, Gaithersburg, Maryland, USA) programs. Validation of extraction, derivatization, and GC-MS analysis was performed for selected mono- and disaccharides and recovery percentage was calculated [15]. Metabolite concentrations were determined as “(analyte concentration relative to hentriacontanoic acid) per 500 μL serum” (unitless relative concentration) (for details see [12]).

For statistical analysis and reporting, the unitless relative concentrations were divided by the molecular weight (MW) of the saccharide. All analyses were carried out using SAS 9.4 (SAS Institute Inc, Cary, North Carolina, USA). To place the subset selection process in perspective, there are 50,389 subsets comprised of 1 to 7 of the 19 saccharides (PROC ALLCOMB). To tie to our previous work [12], and to examine the nest (**B**urrow, **S**urface) × sex (**F**emale, **M**ale) interaction without explicitly partitioning the 30 penguins into 4 small subsets (**F**-**B** = 7, **F**-**S** = 3, **M**-**B** = 10, **M**-**S** = 10), preliminary models included sex as a nominal predictor and nest as the binary response in some analyses. In other analyses, sex was the binary response and nest was the nominal predictor.

Three *preliminary* analyses of all 19 saccharides (sugars + metabolites) were carried out to identify a relevant saccharide subset. First, we identified saccharides with a fold-change $\frac{max}{min}\geq |2|$ between burrow- and surface-nesters. Second, before model-building, data were binned to discriminate burrow and surface nests. PROC HPBIN calculates a “weight of evidence” measure we designate as Ψ as the proportionate amount that an attribute supports or weakens a hypothesis [16] (Eq 1). The values of the target variable (a non-event or an event) determine the value. This analysis supported differences in saccharide profiles between bins (nest locations), so these values were transformed into information values (IV; Eq 2), which are more intuitive (i.e., larger values are better predictors). Third, logistic regression estimates of concordance (*c*), Somers’ D, gamma and tau-a were obtained for the top 222 models from discriminant analyses. When the response is binary, the concordance index *c* is the area under the receiver operating characteristic (ROC) curve [17, 18]. The index *c* varies from 0.5 (no evident accuracy) to 1.0 (complete accuracy).

$$Proportionate\;Amount:\;\Psi =\lfloor ln(\frac{\%\;of\;non-events}{\%\;of\;events})\rfloor \times 100$$ (1)

$${Information\;Value:\;IV}_{x}=\sum_{x=1}^{x=\#\;Bins}({bad}_{i}-{good}_{i})\times \Psi$$ (2)

SAS tabulates Ψ as the bin number. The nest designations (burrow = 0, surface = 1) were added to the Ψ matrix. The simple matching correlation coefficient, S4 = (*a* + *b*)/*N* was calculated for each saccharide (predictor) against nest designation. The same analyses were done for sex (female = 0, male = 1).

We concluded from the *preliminary* analyses that a strategy for identifying best subsets of up to 7 saccharides was concordance of Ψ and IV and then two standard analyses of all-possible-subsets: 1) ANOVA using Proc GLM to estimate effect-size and 2) discriminant analysis for sensitivity and specificity. We defined K as the number of saccharides in a subset. Nineteen subsets had K = 1 saccharide, 3877 subsets had K = 1–4 saccharides, and 50389 subsets had K = 1–7 saccharides. The first filter for subset retention was 0.9 ≤ specificity/sensitivity (S/S) ≤ 1.1, that is max(specificity) ≈ max(sensitivity). A second filter was min(K). Subsets of > 7 saccharides did little to improve discrimination of saccharide profiles between surface- and burrow-nesters.

We included Cohen’s effect size measure *d* [19, 20] and a Winsorized version. The Winsorized version of Cohen’s is $d_{W}=\nu ({\overset{-}{x}}_{1W}-{\overset{-}{x}}_{2W})/{SD}_{Wpooled}$, where ${\overset{-}{x}}_{1W}\;and\;{\overset{-}{x}}_{2W}$ are the Winsorized means, SD_WPooled_ is the pooled standard deviation of the Winsorized data (SD_*W*_), and *v* is a correction factor. The only published value of *v* we found was 0.642 for two-sided 20% (*γ* = 0.2) Winsorization. We estimated *v* = SD_W_/SD_Random_ for two-sided and one-sided trimming of 10% (*γ* = 0.1) and 20% (*γ* = 0.2) using a random sample of *n* = 10^7^ values from a normal distribution with mean = 0 and SD = 1. The number of values trimmed on a side is *g* = *γn* rounded down to the nearest integer. The Winsorized sample replaces the upper *g* trimmed values by the largest value not trimmed and the lower *g* trimmed values by smallest value not trimmed [21].

## Results

Nineteen saccharides were quantified in 30 penguins (Table 1). Thirteen penguins nested on the surface and 17 nested in sheltered burrows or crevices. Ten penguins were female and 20 were male. Saccharide profiles did not differ between Humboldt penguin sexes, and sex was excluded as a predictor (Table 1). Our value of v for two-tailed 20% Winsorization agrees exactly with the published value, 0.642 (Table 2).

**Table 1:** Saccharides identified in 30 free-ranging Humboldt penguins. Concentration relative to standards, mean (SD), and Cohen’s *d* for nest and sex.

**Saccharide, MW, {CASRN}**^a^	**RAW Burrow N = 17**			**WINSORIZED**^**b**^		**RAW Surface N = 13**		**WINSORIZED**^**b**^		
	${\overset{-}{x}}_{1}$	***SD***_**1**_	***d***_***raw***_	${\overset{-}{x}}_{1W}$	***SD***_**1W**_	${\overset{-}{x}}_{2}$	***SD***_**2**_	${\overset{-}{x}}_{2W}$	***SD***_**2W**_	***d***_**W**_
1,6-Anhydro-β-D-glucopyranose, 1,6-Anhydro-β-D-glucose, 1,6-Anhydroglucose, Levoglucosan, 162.14, {498-07-7, 54-17-1}	0.36	0.51	-0.38	*0*.*19*	*0*.*03*	0.64	0.98	*0*.*34*	*0*.*17*	*-0*.*32*
Arabinose, 150.13 {147-81-9}	1.61	3.22	-0.85	*0*.*46*	*0*.*22*	9.70	14.11	*5*.*81*	*4*.*87*	*-1*.*43*
D-Fructose, 180.16 {57-48-7}	4.62	7.06	0.06	*2*.*95*	*2*.*67*	4.23	4.58	*2*.*77*	*1*.*50*	*0*.*66*
D-Galactose, 180.16 {59-23-4}	6852.37	2773.32	0.38	*5890*.*51*	*929*.*35*	6012.56	1111.55	*5817*.*42*	*839*.*33*	*-8*.*82*
D-Glucoheptulose, 210.18 {5349-37-1}	6.86	7.69	0.35	*4*.*44*	*2*.*13*	4.70	3.18	*4*.*11*	*2*.*44*	*7*.*63*
D-Glucopyranose, 180.16 {54-17-1}	1486.62	430.92	-0.50	*1388*.*07*	*301*.*99*	1727.33	538.44	*1672*.*90*	*486*.*60*	*-4*.*36*
D-Glucose, 180.16 {50-99-7}	23060.38	7177.13	-0.36	*20200*.*29*	*2284*.*65*	25315.92	4519.72	*24789*.*83*	*4388*.*73*	*-6*.*65*
D-Maltose, 342.3 {69-79-4}	32.74	19.56	0.48	*26*.*18*	*9*.*55*	24.38	13.62	*21*.*01*	*8*.*69*	*6*.*22*
D-Mannose, 180.16 {3458-28-4}	23.29	17.24	0.15	*18*.*55*	*9*.*72*	21.06	11.02	*19*.*73*	*9*.*09*	*0*.*12*
D-Ribose, 150.13 {50-69-1}	2.66	1.60	-0.58	*2*.*36*	*0*.*93*	4.58	4.72	*4*.*15*	*4*.*12*	*1*.*76*
D-Xylose, 150.13 {58-86-6}	0.66	1.02	-0.43	*0*.*22*	*0*.*06*	2.23	5.39	*0*.*58*	*0*.*37*	*0*.*95*
Fructose 6-phosphate, 260.14 {643-13-0}	0.46	0.69	-0.82	*0*.*19*	*0*.*02*	1.14	0.99	*0*.*95*	*0*.*71*	*-0*.*57*
Galactofuranose, 180.16 {19217-07-3}	29.55	21.86	-0.15	*21*.*85*	*7*.*06*	32.48	14.37	*29*.*80*	*10*.*79*	*-3*.*39*
Glucose 6-phosphate, 260.14 {56-73-5}	0.81	0.71	-0.69	*0*.*65*	*0*.*41*	1.40	1.01	*1*.*21*	*0*.*76*	*2*.*91*
Levoglucosenone^c^, 126.11 {37112-31-5}	51.99	28.07	-0.23	*44*.*81*	*17*.*58*	59.28	36.60	*54*.*25*	*29*.*68*	*-2*.*27*
N-Acetylglucosamine, 221.21 {7512-17-6}	17.64	12.67	0.54	*14*.*19*	*6*.*67*	11.62	8.92	*9*.*08*	*5*.*08*	*1*.*66*
Sedoheptulose (D-altro-2-heptulose), 210.18 {3019-74-7}	1.11	1.94	-0.04	*0*.*31*	*0*.*24*	1.17	1.07	*0*.*95*	*0*.*67*	*1*.*75*
Sedoheptulose 7-phosphate, 290.16 {2646-35-7}	1.85	1.17	-0.13	*1*.*56*	*0*.*69*	1.99	0.97	*1*.*78*	*0*.*64*	*-1*.*04*
Sucrose, 342.3 {57-50-1}	0.41	0.45	-0.46	*0*.*27*	*0*.*17*	2.52	6.96	*0*.*58*	*0*.*53*	*1*.*67*
**Saccharide, MW, {CASRN}**^a^	**RAW FEMALE N = 10**			**WINSORIZED**^**b**^		**RAW MALE N = 20**		**WINSORIZED**^**b**^		
	${\overset{-}{x}}_{1}$	***SD***_**1**_	***d***_***raw***_	${\overset{-}{x}}_{1W}$	***SD***_**1W**_	${\overset{-}{x}}_{2}$	***SD***_**2**_	${\overset{-}{x}}_{2W}$	***SD***_**2W**_	***d***_**W**_
1,6-Anhydro-β-D-glucopyranose, 1,6-Anhydro-β-D-glucose, 1,6-Anhydroglucose, Levoglucosan, 162.14, {498-07-7, 54-17-1}	0.27	0.24	-0.40	*0*.*20*	*0*.*00*	0.57	0.90	*0*.*31*	*0*.*17*	*-0*.*65*
Arabinose, 150.13 {147-81-9}	2.22	5.35	-0.43	*0*.*36*	*0*.*23*	6.57	11.82	*4*.*17*	*4*.*44*	*-0*.*87*
D-Fructose, 180.16 {57-48-7}	2.29	1.91	-0.55	*1*.*71*	*0*.*77*	5.54	7.06	*4*.*14*	*3*.*66*	*-0*.*66*
D-Galactose, 180.16 {59-23-4}	6192.16	1427.80	-0.19	*5788*.*15*	*706*.*39*	6628.68	2555.44	*5858*.*32*	*937*.*91*	*-0*.*07*
D-Glucoheptulose, 210.18 {5349-37-1}	5.16	2.65	-0.17	*4*.*70*	*2*.*02*	6.23	7.42	*4*.*16*	*2*.*44*	*0*.*19*
D-Glucopyranose, 180.16 {54-17-1}	1405.20	392.45	-0.56	*1315*.*76*	*252*.*82*	1674.95	523.52	*1632*.*59*	*470*.*27*	*-0*.*64*
D-Glucose, 180.16 {50-99-7}	20945.98	4946.62	-0.74	*19035*.*21*	*2177*.*40*	25416.94	6475.52	*24127*.*57*	*4080*.*35*	*-1*.*18*
D-Maltose, 342.3 {69-79-4}	23.79	14.55	-0.47	*19*.*01*	*4*.*63*	31.87	18.44	*28*.*15*	*12*.*61*	*-0*.*71*
D-Mannose, 180.16 {3458-28-4}	23.96	16.16	0.13	*21*.*99*	*14*.*97*	22.10	13.41	*19*.*46*	*8*.*91*	*0*.*19*
D-Ribose, 150.13 {50-69-1}	3.00	3.37	-0.22	*2*.*02*	*1*.*09*	3.76	3.46	*3*.*42*	*2*.*89*	*-0*.*47*
D-Xylose, 150.13 {58-86-6}	0.34	0.30	-0.42	*0*.*19*	*0*.*03*	1.85	4.40	*0*.*77*	*0*.*72*	*-0*.*81*
Fructose 6-phosphate, 260.14 {643-13-0}	0.40	0.63	-0.60	*0*.*20*	*0*.*00*	0.92	0.96	*0*.*78*	*0*.*69*	*-0*.*84*
Galactofuranose, 180.16 {19217-07-3}	29.36	20.67	-0.13	*22*.*93*	*6*.*59*	31.84	17.92	*29*.*11*	*13*.*25*	*-0*.*45*
Glucose 6-phosphate, 260.14 {56-73-5}	0.60	0.52	-0.84	*0*.*51*	*0*.*36*	1.30	0.95	*1*.*17*	*0*.*75*	*-0*.*84*
Levoglucosenone^c^, 126.11 {37112-31-5}	59.73	33.57	0.22	*55*.*14*	*27*.*68*	52.73	31.46	*47*.*46*	*23*.*16*	*0*.*26*
N-Acetylglucosamine, 221.21 {7512-17-6}	14.63	6.08	-0.07	*12*.*50*	*4*.*28*	15.44	13.31	*12*.*38*	*8*.*03*	*0*.*01*
Sedoheptulose (D-altro-2-heptulose), 210.18 {3019-74-7}	1.18	2.28	0.04	*0*.*42*	*0*.*43*	1.12	1.19	*0*.*91*	*0*.*82*	*-0*.*57*
Sedoheptulose 7-phosphate, 290.16 {2646-35-7}	1.20	0.81	-1.17	*0*.*96*	*0*.*43*	2.30	0.99	*2*.*07*	*0*.*66*	*-1*.*56*
Sucrose, 342.3 {57-50-1}	0.26	0.34	-0.34	*0*.*15*	*0*.*08*	1.86	5.62	*0*.*59*	*0*.*50*	*-0*.*88*

^a^ The Chemical Abstracts Service (CAS), http://www.cas.org/, is a division of the American Chemical Society, https://www.acs.org/.

^c^ Code by R Wicklin, How to Winsorize data in SAS (The DO Loop July 15, 2015 https://blogs.sas.com/content/iml/2015/07/15/winsorize-data.htm).

^c^ Levoglucosenone is from exogenous sources (see text).

**Table 2:** Scaling factors for the ratio of Winsorized standard deviations estimated using 10^7^ random values from a normal distribution with mean = 0 and SD = 1.

	Mean	Std Dev	Minimum	Maximum	Adjustment
Random sample	5.38E-05	1.00008	-5.0722082	5.2492638	
γ = 0.2, 2-sided	8.67E-05	0.64192	-0.8415472	0.8414321	0.642
γ = 0.2, Upper 1-sided	-1.12E-01	0.83291	-5.3790776	0.8414321	0.833
γ = 0.2, Lower 1-sided	1.12E-01	0.83278	-0.8415472	5.5836618	0.833
γ = 0.1, 2-sided	9.71E-05	0.82400	-1.2820395	1.2819490	0.824
γ = 0.1, Upper 1-sided	-4.72E-02	0.91511	-5.3790776	1.2819490	0.915
γ = 0.1, Lower 1-sided	4.74E-02	0.91500	-1.2820395	5.5836618	0.915

Four saccharides had fold-change (FC) $\frac{max}{min}\geq 2$: arabinose (6.0), D-xylose (3.4), fructose 6-phosphate (2.5), and sucrose (6.2). The fold-change for 1,5-anhydroglucose was 1.73. The distributions of *c*, Somers’ D, gamma, and tau-a from logistic regression of 222 models were bimodal. Concordance ranged from 0.88 to 0.93, Somers’ D and gamma from 0.76 to 0.86, tau-a from 0.39 to 0.43, and all R_Spearman_ = 1.0. Thus, these measures did not discriminate among the models.

Together, the top 100 subsets from discriminant analysis included all 19 saccharides (Table 3). The top 5 subsets included arabinose, D-glucopyranose, D-glucose, D-maltose, galactofuranose, glucose 6-phosphate, and levoglucosenone. Five saccharides in the top 10 subsets were not in the top 5 subsets: D-galactose, D-xylose, fructose 6-phosphate, N-acetylglucosamine, and sedoheptulose. D-Ribose had the highest information value (IV), followed by glucose 6-phosphate, levoglucosenone, and D-galactose (Table 4). D-maltose, galactofuranose, 1,6-anhydroglucose, D-fructose, sedoheptulose, and sedoheptulose 7-phosphate had no information value. The other saccharides had IV values of intermediate or weak [16].

**Table 3:** Saccharides in the top 10 models based on K and the magnitudes (closeness to 100%) and equality of specificity and sensitivity.

K	Saccharides	Specificity (%) (n = 17)	Sensitivity (%) (n = 13)
3 [1]	Arabinose, D-Glucose, D-Maltose	82.35	84.62
3 [2]	D-Maltose, Glucose-6-phosphate, Levoglucosenone	82.35	84.62
4 [3]	Arabinose, D-Glucopyranose, D-Maltose, Levoglucosenone	94.12	92.31
4 [4]	Arabinose, D-Maltose, Galactofuranose, Levoglucosenone	88.24	92.31
4 [5]	Arabinose, D-Maltose, Glucose-6-phosphate, Levoglucosenone	88.24	92.31
4 [6]	Arabinose, D-Xylose, Levoglucosenone, Sedoheptulose	88.24	92.31
4 [7]	Arabinose, D-Galactose, D-Maltose, D-Xylose	88.24	84.62
4 [8]	Arabinose, D-Galactose, D-Maltose, Fructose-6-phosphate	88.24	84.62
4 [9]	Arabinose, D-Glucopyranose, Levoglucosenone, N-Acetylglucosamine	88.24	84.62
4 [10]	Arabinose, D-Glucose, D-Maltose, D-Xylose	88.24	84.62

**Table 4:** Frequency of occurrence in the top 100 models, and for each predictor (saccharide), the proportionate amount Ψ^a^ compared to nest using the S4 matching correlation [*(a+d)/N*]. Sort order is decreasing information value (IV) of the predictor.

Saccharide	Top 100 models [Rank], Frequency, (% of 438 occurrences)	Ψ (S4 positive matching correlation = *(a+d)/N*	Information value (IV)
*Strong predictive power*^b^			
D-Ribose	[18.5] 6 (1.35%)	0.733	1.054
Glucose 6-phosphate	[9.5] 21 (4.71%)	0.700	0.640
Levoglucosenone	[4] 68 (15.25%)	0.667	0.395
D-Galactose	[8] 23 (5.16%)	0.467	0.330
*Medium-weak predictive power*^b^			
Fructose 6-phosphate	[12] 19 (4.26%)	0.633	0.229
D-Glucose	[6] 31 (6.95%)	0.633	0.223
D-Mannose	[18.5] 6 (1.35%)	0.467	0.207
N-Acetylglucosamine	[4] 68 (15.25%)	0.467	0.207
D-Glucoheptulose	[17] 8 (1.79%)	0.500	0.173
Arabinose	[2] 69 (15.47%)	0.600	0.111
D-Xylose	[11] 20 (4.48%)	0.600	0.111
Sucrose	[4] 68 (15.25%)	0.600	0.111
D-Glucopyranose	[7] 25 (5.60%)	0.600	0.106
D-Maltose	[1] 77 (17.26%)	0.500	0.043
Galactofuranose	[15] 12 (2.69%)	0.533	0.019
1,6-Anhydroglucose	[14] 15 (3.32%)	0.567	0.005
D-Fructose	[16] 10 (2.24%)	0.567	0.005
Sedoheptulose	[13] 17 (3.90)	0.567	0.005
Sedoheptulose 7-phosphate	[9.5] 21 (4.71%)	0.533	0.000

^a^ To minimize confusion of the meaning of “weight of evidence (WOE)” as used in statistical analysis from WOE as used in regulations and industry, we use and define the symbol Ψ in Eq 1

^b^ Rules of thumb for IV values: strong IV > 0.3; Medium 0.1 ≤ IV ≤ 0.3; weak 0.2 ≤ IV ≤ 0.1; None IV ≤ 0.2 [16 p. 76]

## Discussion

The Humboldt penguin is specialized for foraging in the highly productive cold-water upwellings of the Humboldt Current. The penguins may travel long distances to find productive regions for foraging and subsist on a diet of primarily Peruvian anchovies (*Engraulis ringens*) and other pelagic forage fish. The exact diet composition of the animals in this study is unknown, but is postulated to be consistent amongst the cohort given the temporal clustering of sample collection over 5 days with static ocean conditions. The most recent active foraging time relative to blood collection is also unknown. This lack of data does present a minor confounding variable, but is unavoidable when working with wild penguins in free-ranging ocean environments. Known metabolic pathways for healthy humans, non-human vertebrates, and *Escherichia coli* may be relevant for the evaluation of penguin results, as are pathways in fasting birds (penguins [22, 23]) and female mallards (*Anas platyrhynchos* [24]) and hibernating vertebrates [25–27]. The major pathways of carbohydrate and lipid metabolism in birds and mammals are qualitatively similar but differ quantitatively in the relative activities of some pathways and individual enzymes [28–30]. Pollock reviewed some clinical implications of glucose metabolism in birds [30].

Although male Humboldt penguins are larger than females, there is such a considerable degree of overlap in size that discriminant function analysis of measurements is required to determine sex with 95% accuracy [31, 32]. While some metabolites in this study revealed statistically significant differences between sexes, no saccharide profiles were different between sexes. Given these similarities in body size and shared parental roles, the observed lack of sex-based difference in the saccharide profiles of the penguins in this study is expected and consistent with other measures of energy expenditure in penguin species. Differences in individual metabolites may be related to sample size constraints or clinically irrelevant factors. In the similarly sized gentoo penguin (*Pygoscelis papua*), another monomorphic species with shared parental duties, daily energy expenditures throughout the breeding season is similar in both sexes [33]. In Adélie penguins, (*Pygoscelis adeliae*), both sexes lose mass (depot fat and lean tissue) during the early breeding season, but males have a greater cumulative reproductive effort, which could suggest that differences in energy metabolism may exist in some species. Breeding Adélie penguins feed chicks according to their condition and food availability [34], and adults in better condition at the outset lose a greater proportion of mass [35]. When food is limiting, adults bring less food to chicks in order to maintain their body mass.

Univariate and multivariate statistical analyses identified serum saccharides (in particular, arabinose, maltose, glucose-6-phosphate, and levoglucosenone) in Humboldt penguins that were nest-dimorphic with substantial differences between surface- and sheltered-nesting penguins. Circulating metabolites come from endogenous processes (e.g., mitochondrial metabolism) and exogenous sources including food [36] and intestinal *E*. *coli* [37–38]. With large sample sizes, the likelihood increases that a difference that is not biologically or practically relevant will be detected statistically, thus overstating the differences in the data-set. An editorial by the Executive Director of the American Statistical Association and colleagues stated: “Thoughtful research looks ahead to prospective outcomes in the context of theory and previous research. Researchers would do well to ask, *What do we already know*, *and how certain are we in what we know*? And building on that and the field’s theory, *what magnitudes of differences*, *odds ratios*, *or other effect sizes are practically important*? These questions would naturally lead a researcher, for example, to use existing evidence from a literature review to identify specifically the findings that would be practically important for the key outcomes under study” [39]. In the case of our analysis, seventeen burrow-nesters and thirteen surface-nesters are small sample sizes [40, 41], so the significant differences in the saccharide profiles are likely to be correct. Causality and biological relevance of a significant nest difference for all saccharides are not obtainable from our data.

Of the seven saccharides in the top five predictive modeling combinations, 4 are simple sugars (D-glucopyranose, α ⇄ β; D-glucose; D-maltose; and D-mannose) and three are derivatives (glucose 6-phosphate, levoglucosenone, and N-acetylglucosamine). As reported in over 30 papers, glucose metabolism has been studied in several penguin species under various conditions and durations of feeding, breeding, and starvation [42–51]. Glucose metabolism in penguins links to well-described pathways in other vertebrates (including other *Aves*). Whole blood samples were chilled upon collection to slow cellular metabolism, and serum was separated within 6 hours. Since nucleated avian erythrocytes do not utilize plasma glucose like mammalian cells, variation in the time between collection and centrifugation was not recorded or analyzed.

Glucose regulates lipid metabolism in penguins and affects hormonal status differently in stressed and non-stressed individuals [52]. For example, stress due to prolonged fasting is characterized by protein sparing and decreased energy expenditure, whereas stress due to molt is distinguished by increased catabolism of body protein and energy expenditure [53]. Plasma glucose (mean ± SEM mmol/L) concentrations and glucose mass (mmol/kg) are not statistically different in emperor penguins during feeding, fasting during breeding, and forced fasting, but fractional glucose turnover rate (%/min) is 38% greater in the fed than the fasted state. These concentrations are notably less than those observed in fasting penguins during molting (28.2 ± 6.1 [43]). In penguins, as in other fasting vertebrates, muscle proteins are used to provide blood glucose for maintenance of the central nervous system during fasting and glucogenic amino acids for oxaloacetate formation [54]. Similar to glucose, arabinose, glucose 6-phosphate, and maltose are higher in serum from surface-nesting penguins, possibly signifying greater catabolism of muscle.

Maltose is a glucose dimer. Metabolism and transport processes for maltose are conserved from bacteria to humans [55], and so lower organisms are models for maltose chemistry and biochemistry in higher organisms [56]. Complete digestion of starch (by gut bacteria) produces maltose that is then hydrolyzed by maltase to two molecules of glucose. Gastrointestinal microbiota in penguins, as in other vertebrate species, are dominated at the phylum level by Bacteroidetes and Firmicutes, although the composition of the fecal microbiota varies among penguin species [57]. Maltose may help regulate satiety in penguins by suppressing appetite in a concentration-dependent manner that is complementary to dose-dependent appetite suppression seen in monkeys under experimental conditions [58]. Appetite suppression in surface-dwelling penguins would conserve energy by reducing the number of feeding trips made to satisfy satiety.

The PSJ peninsula, where this research was conducted, protects the largest breeding colony of Humboldt penguins in Peru (3,000–5,000 penguins). This colony is 25–50% of the entire Peruvian population and up to 15% of the global population on an annual basis [7, 1], making the protection of PSJ paramount for species conservation. As previously discussed, historical commercial harvesting of guano had devastating effects on seabird populations around the globe. Peruvian efforts to maximize guano production in the early 1900s led to the creation of a network of guano reserves, including PSJ, that were artificial islands with physical barriers separating a natural peninsula from human disturbance and predator intrusion as a means of protecting nesting populations of cormorants, pelicans, and boobies. While these reserves protected substantial flighted seabird populations, Humboldt penguin populations were adversely affected by the harvesting activities that removed nesting sites and substrate [8]. Legislation in 2009 transitioned oversight of the marine network to the Peruvian Ministry of the Environment, which now requires guano harvesting to be conducted in a sustainable manner that leaves guano deposits behind in designated areas for penguin nesting habitat. Suboptimal surface-nesting locations may result in penguins expending additional energy to maintain a suitable nest microhabitat for incubation, protect the eggs and chicks from predators, and tolerate intense exposure to wind and sun. This increased energetic demand is a highly plausible explanation for the differences in saccharide profiles.

Previous investigation of environmental contaminants in penguins at PSJ found low contaminant levels and population health concerns [13, 59]; however, the current data allowed for further evaluation of select contaminants based on nesting type. Burning of cellulose produces tri-hydroxylactone levoglucosan (1,6-anhydro-β-D-glucopyranose), and further dehydration produces the unsaturated ketone levoglucosenone (1,6-anhydro-3,4-dideoxy-β-D-glycero-hex-3-enopyranos-2-ulose) [60]. Levoglucosan is the most abundant carbohydrate in haze aerosols from smoldering fires and is a suggested tracer for biomass burning [61–64]. Chemical analysis of particulate matter from combustion samples has shown that levoglucosan is an unequivocal biomass burning tracer [61].

The number of understory fires in the southern Amazonia region that includes southeastern Peru dramatically increased in 2005 and 2007, when 14,000 and 26,000 km^2^ burned [65]. Also, fires are regularly set in montane areas of the Peruvian Andes to clear woody vegetation and stimulate vegetation for grazing [66, 67]. The presence of levoglucosenone in the serum of all penguins examined and its higher relative concentration in surface-nesting penguins is especially interesting.

Levoglucosenone in serum was unequivocal evidence of exogenous contamination in the nesting environment of Humboldt penguins at PSJ in 2009 when samples were collected. The presence of levoglucosan in the penguins shows that marine and terrestrial species are exposed to [68], and adsorb [63], combustion-related toxicants whose health effects are unknown. Most ingested levoglucosan is excreted quickly and unchanged [69, 70], which would indicate low toxicity. However, levoglucosan and levoglucosenone initiated skin tumors in 20% and 10–25% of mice, respectively [71]. Recent cytotoxic assays reported that levoglucosenone was active against the pathogen *Salmonella enterica* serovar *typhimurium* (strain MS14028s), and was cytotoxic against CHO cells and human hepatocarcinoma lines (Huh-7, IC_50_ = 12.95 μM; HepG2, IC_50_ = 39.39 μM) [72]. It also was biocidal to Gram (−) and Gram (+) bacteria [73].

The health effects of wood smoke in Humboldt penguins have not been reported, but there is a chain of evidence that some effects are not benign, and some may be sex-related. For example, exposure of humans to wood smoke altered inflammatory responses to viral infection in a sex-specific manner [74]. KEGG analyses of four *Pygoscelis* penguin species detected many essential genes involved in the major innate immunity pathways, which are critical metabolic pathways for maintaining homeostasis against exogenous infections or toxins [75]. Furthermore, blood and liver tissues from urban and rural populations of great tits (*Parus major*) had substantial differences in gene expression profiles based on environment, with differentially expressed genes functioning in roles related to immune and inflammatory responses, detoxification, protection against oxidative stress, lipid metabolism, and regulation of gene expression. Many genes linked to stress responses were expressed at higher levels in the urban birds [76]. Plasma corticosterone responses to handling were similar in great tit and Adélie penguin [77], and Magellanic penguin chicks exposed to tourists had elevated corticosterone stress responses relative to undisturbed chicks in the same breeding colony [78]. In phase II fasting status king penguins under field conditions, noise from human activity affected glucose regulation of lipid metabolism and decreased plasma glucagon 6.5-fold and corticosterone 2.8-fold [52]. We project similar differences in gene expression between Humboldt penguins in unsheltered and sheltered nests.

## Conclusions

Statistical analysis identified differences in saccharide profiles of surface- and sheltered-nesting Humboldt penguins that likely reflect increased metabolic requirements of surface-nesters. Ramifications of increased metabolic demand warrant further investigation as this requirement is proposed to contribute to decreased reproductive success. Surface-nesting penguins also have higher serum concentrations of levoglucosenone, which is likely indicative of greater exposure to environmental contamination via the burning of land biomass. Conservation strategies that preserve burrowing substrate as essential habitat for nesting penguins are warranted and should be continued.

## Supporting information

S1 Data (XLSX)
